# Effect of antenatal depression on ANC service utilization in northwest Ethiopia

**DOI:** 10.1038/s41598-023-37382-9

**Published:** 2023-09-02

**Authors:** Getnet Mihretie Beyene, Telake Azale, Kassahun Alemu Gelaye, Tadesse Awoke Ayele

**Affiliations:** 1 Department of Psychiatry, College of Health Sciences, Debre Tabor University, Debre Tabor, Ethiopia; 2 Department of Health Education and Behavioral Sciences, Institute of Public Health, College of Medicine and Health Sciences, University of Gondar, Gondar, Ethiopia; 3 Department of Epidemiology and Biostatistics, Institute of Public Health, College of Medicine and Health Sciences, University of Gondar, Gondar, Ethiopia

**Keywords:** Human behaviour, Health care, Risk factors, Psychiatric disorders

## Abstract

Maternal morbidity and mortality remain high among women who did not attend antenatal care (ANC). Antenatal care is one of the interventions given to pregnant women to detect existed problems or problems that can develop during pregnancy, which harm the health of pregnant women and fetuses. In Ethiopia, however, there is limited evidence that revealed the effect of antenatal depression on ANC service utilization. Hence, this study aimed to see the effect of antenatal depression on ANC visits among women in urban northwest Ethiopia. A population-based, prospective cohort study was done from June 2019 to March 2020. The Edinburgh postnatal depression scale was administered to 970 women in the second and third trimesters of pregnancy to screen for antenatal depression. Additional data were collected on ANC visits, the mother’s socio-demographic, obstetric, clinical, psychosocial, and behavioral factors. A logistic regression model was used to adjust confounders and determine associations between antenatal depression and inadequate ANC visits. The cumulative incidence of inadequate ANC visits was 62.58% (95% CI: 59.43, 65.63). The cumulative incidence of inadequate ANC visits among depressed pregnant women was 75% as compared to 56% in non-depressed. The incidence of inadequate ANC visits in the exposed group due to antenatal depression was 25.33%. After multivariable analysis, antenatal depression at the second and third trimesters of pregnancy remained a potential predictor of inadequate ANC visits (AOR = 1.96: (95% CI 1.22, 3.16)). In addition, antenatal depression, long travel time for ANC visits (AOR = 1.83 (95% CI 1.166, 2.870)), and late initiation of ANC visits (AOR = 2.20 (95% CI 1.393, 3.471)) were the predictors of inadequate ANC visits as compared to their counterpart. This study suggested that antenatal depression affects ANC visits in Ethiopian urban settings. Therefore, early detecting and treating depression symptoms during the antenatal period reduced significantly the impacts of depression on the health of the mother and fetus.

## Introduction

Maternal well-being and survival are not only crucial to the health of the mother but also to the newborn’s health and development. The global maternal mortality ratio(MMR) estimate in 2020 was 152 maternal deaths per 100,000 live births from complications arising from pregnancy and childbirth^1^. The lifetime risk of maternal mortality due to pregnancy complications is higher in Sub-Saharan Africa than in other parts of the world; because of inaccessibility to ANC clinics, risk of poverty, and HIV infection^2^. In Ethiopia, despite the existence of interventions that could prevent maternal morbidity, death, and disability during pregnancy and childbirth, the levels of maternal mortality are the highest in the world. In Ethiopia, the maternal mortality ratio from preventable pregnancy-related complications remains high (255 per 100,000 live births) until 2020^1^.

Antenatal care is the health care given to pregnant women to detect problems that existed before or that occur during pregnancy, which harm the health of pregnant women and the fetuses^3^. To maintain a healthy pregnancy for mother and baby, women required a positive pregnancy experience (including preventing or treating risks, illness, and death) with good quality antenatal care^4^. Studies showed that maternal antenatal care follow-up during pregnancy is a significant determinant of maternal morbidity and mortality^5^. Timely and appropriate antenatal care (ANC) visits are the cornerstone to reduce morbidity and mortality of pregnant women and newborns^3,5^. A cohort study revealed that, having four or more ANC visits reduced postpartum haemorrhage, early neonatal death, preterm labour, and low birth weight by 81.2%, 61.3%, 52.4%, and 46.5%, respectively^6^.

World Health Organization (WHO) recommended a minimum level of antenatal care visits should consist of four visits in one pregnancy to reduce the risk of pregnancy and obstetric complications^7–9^. Globally, 85% of pregnant women attend at least one ANC visits with a skilled health professional and 58% attend at least 4 ANC visits^10^; in Ethiopia, the Mini Demographic Health Survey (EMDHS) 2019 reported that the proportion of pregnant women who attended four or more ANC visits was only 43%^11^. The research finding indicates that antenatal care services are available to all pregnant women in low and middle-income countries with high-quality and minimal cost or no cost program to encourage adequate ANC use^12^, but studies in Yemen^13^ and Ethiopia^14,15^ revealed that there is only a small number of pregnant women receiving four or more antenatal care visits. These acts are alarming to examine the critical factors influencing the uptake of ANC services. Marital status, maternal age, lack of time to attend, and women’s economic status were significant predictors of utilization of maternity care^16^. Education status, marital status, residence, parity, and religion were potential determinants of health care utilization^17^. Other important factors that affected the utilization of maternity care services were: the high cost of antenatal care services, long waiting times, and poor staff attitude^13^. A study done in Rwanda showed, the age of participants and poor social support were predictors of underutilization of ANC services^18^.

Currently, there is increasing evidence that revealed antenatal depression is a significant public health problem in low- and middle-income countries (LAMICs)^19^. Depression is among the most prevalent mental health problems during pregnancy, affecting about one in four women during their lifetime^20^. The adverse effects of antenatal depression on prolonged labor, peripartum and postpartum complications, non-vaginal delivery, and newborn illness have been well documented^21^. Antenatal depression has a negative effect on ANC service utilization and thereby contributes to increased perinatal complications and maternal mortality. Studies conducted in the USA showed that antenatal depression was significantly associated with poor prenatal care utilization^22–24^. There is also evidence from high-income settings that antenatal depression is associated with inadequate antenatal care^25^. A study in the East Asia region revealed that antenatal depression negatively affected pregnant women's antenatal care visits^26^. On the contrary, a study done in Minnesota revealed that inadequate prenatal care utilization was significantly associated with a past psychiatric history, but not with a current antenatal psychiatric diagnosis^27^. Studies in Africa showed that no association was found between antenatal depression and antenatal care attendance^21,28^.

In Ethiopia, a population-based study indicated that pregnant women with depressive symptoms had an increased risk of having more non-scheduled ANC visits, but not with the adequacy of ANC visits^29^. However, studies examining the effect of antenatal depression on ANC visits have been scanty in number. There is a clear gap that showed the effect of antenatal depression on antenatal care use, particularly in Ethiopia where maternal mortality is high. Therefore, this study aimed to explore the effect of antenatal depression on ANC visits in northwest Ethiopia.

## Methods

### Study design and settings

We conducted a community-based prospective cohort study at Debre Tabor and Woreta towns from June 2019 to March 2020 in the South Gondar zone, northwest Ethiopia. According to the South Gondar zonal catchment profile, the estimated total population of Debre Tabor town was 84,382, of which 40,753 are females. While Woreta town has an estimated population of 41,668, of which 20,507 are females. The estimated number of pregnant women per year in Debre Tabor and Woreta towns were 2844 and 1404 respectively^30^. In these towns, the individuals obtain basic health care services from one government-operated referral hospital, five health centers, and ten private health institutions during the data collection period.

Health Extension Workers (HEWs) are responsible for performing prevention and promotion activities, identifying and monitoring pregnant mothers, and maintaining up-to-date maternal records in each Kebele (the lowest administrative unit or village in Ethiopia). The previous year's report of the district health office showed that the proportions of pregnant women who were using antenatal care services at Debre Tabor and Woreta towns were estimated to be 75% and 64%, respectively^30^.

### Sample size

The sample size was estimated using the double population proportion formula by considering the exposed and non-exposed ratio of 1:2 (exposed are those who are depressed, and non-exposed are those who are not depressed), 95% level of confidence, 80% power, and 10% non-response rate.

### Cohort recruitment and eligibility

**Figure 1:**
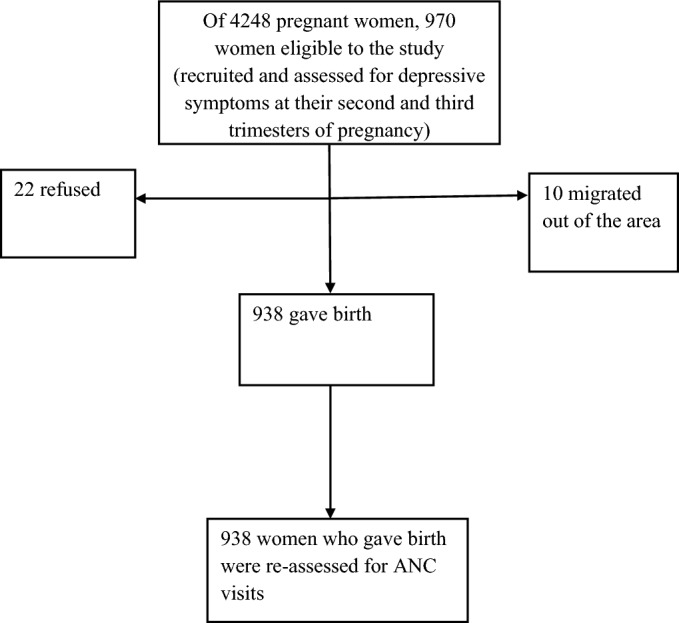
Flow chart of recruited pregnant women and the outcome of inadequate ANC visits.

All eligible and consenting women were recruited into the cohort. Pregnant women who were in their 2nd and 3rd trimesters, living in Debre Tabor and Woreta towns for at least the preceding six months, and without any cognitive or hearing impairment during the study period were eligible for this study. The data collectors were interviewed through home-to-home visits and declared untraceable after three recruiting visits considered as unsuccessful. Totally, 970 women were identified within five months (June 2019–October 2019) and followed up to March 2020. Potentially all cohort pregnant women who gave birth were eligible (Fig. 1).

### Data quality management

A data collection tool was pilot tested in a similar context. The data collectors were Diploma and BSc holders in nursing who had experience in data collection in the field. Before the commencement of data collection, the data collectors and supervisors took comprehensive training with practical exercises for two days that help them to understand the contents of the questionnaire, objectives, and ethical issues relevant to the study. Similarly, Master’s degree holder supervisors were assigned during data collection. Besides, the principal investigator, field supervisors made regular on-site supervision during the data collection periods. The questionnaires were rechecked by the supervisors and the principal investigator for completeness and consistency in a weekly based face to face meetings and telephone communications. Accordingly, incomplete or incorrectly filled questionnaires were identified and managed. For the data entry, templates with proper check codes were designed and employed to minimize data entry errors. The experts who did the data entry had also ample experience on using the Epi-data software. Besides, double entry of the data, 10% of the questionnaires were done to detect and manage the mistakes likely to occur during data entry. Also, the principal investigator randomly cross-checked the correctness of the data entered to the Epi-data software and the data captured in the questionnaire.

### Measurement

#### Primary exposure

Antenatal depression was the primary exposure variable; which was assessed using the EPDS. The EPDS has a sensitivity, specificity, and internal reliability Cronbach's alpha of 78.9%, 75.3%, and 0.71 respectively; from a validation study in Ethiopia^31^. It includes ten items with a Likert scale of responses scored from 0 to 3 to a maximum score of 30; which was then coded as Yes^1^ if there is depression (scores ≥ 12) and No (0) if otherwise (< 12 scores) to indicate scores that are likely to suggest depressive disorder.

EPDS is a preferable scale over other depression scales to screen for depression during pregnancy; because it removes the physical symptoms of depression associated with pregnancy^32^. In these local kebeles, a woman with suicidal ideation was referred to a psychiatric unit in Debre Tabor Hospital for further assessment and appropriate treatment.

#### Outcome variables

The primary outcome variable was inadequate ANC visits. ANC visits were categorized as inadequate (< four visits) and adequate (≥ four visits) visits. ANC visits were obtained by interviewing women after delivery^33^.

#### Potential confounding variables

The most common potential confounding factors were long travel time for ANC visits (categorized as ‘Yes’ if the time takes greater than or equal to 30 min, or ‘No’ if otherwise), threatening life events, lack of time for ANC visit, social support, intimate partner violence, waiting a long time for ANC service (categorized as ‘Yes’ if the time takes greater than or equal to 120 min or ‘No’ if otherwise, initiation of ANC visit (If a mother starts ANC visit within 3 months of pregnancy period = early initiation). In addition, any chronic medical condition, integration of ANC influence ANC service, history of complications current pregnancy, history of alcohol misuse, socio-demographic and economic variables, including marital status, wealth index, and level of education also assessed.

Experiences of stressful life events during the six months before the assessment was assessed using the List of Threatening Experiences (LTE). The scale contains twelve items and includes questions about death, illness, conflicts, and loss of property^34^. The presence of LTE ascertained by the presence of at least one or more LTE. The test–retest reliability of the LTE was good, with a Kappa of 0.61–0.87^35^.

The Oslo Social Support Scale (OSSS-3)^36^ was used to measure maternal social support during pregnancy. The level of social support classified as “poor support”^3–8^, “moderate support”^9–11^, and “strong support”^12,14^ scores. The OSSS-3 consists of three items assessing the number of close intimates, perceived level of concern from others, and perceived ease of getting help from neighbors. The OSSS-3 has good convergent and predictive validity^37^. Both the LTE-12 and the OSSS-3 have been used in a population-level study in Ethiopia^38^.

Intimate Partner Violence (IPV) during pregnancy was assessed by asking three questions related to emotional, physical, and sexual violence. The presence of IPV was ascertained by the presence of at least one type of IPV^39^. The history of physician-diagnosed chronic medical conditions, including cardiac disease, hypertension counted for each woman, recorded as “No” for those without any chronic medical conditions, and “Yes” otherwise History of current pregnancy complications: anemia, hypertension, edema, Antepartum haemorrhage (APH) were counted for each woman and recorded as “yes/no.” Alcohol use was assessed using the four-item scale; the Fast Alcohol Screening Test (FAST)^40^, which ranges from 0 to 16. Hazardous drinking refers to a score of three or more on a FAST scale^40^.

### Analysis strategy

We used Stata software (version 14) for analysis. We run descriptive statistics for all study variables. Percentage values, with their corresponding 95% confidence intervals were used to summarize categorical variables. We used the odds ratio to measure the effect of antenatal depression on ANC visits. We carried out univariate logistic regression analyses for each risk factor to identify possible predictors associated with inadequate ANC visits. During univariate analysis, variables were selected for multivariable analysis if the *p*-value was < 0.2. The difference was deemed to be significant if *p* < 0.05. The total number of losses to follow up was 32 (3.2%). We also used a complete case analysis as it was suggested less than 5% lost to follow-up was with little concern^41,42^. We used a principal component analysis for the wealth index and checked its assumption of whether it fulfills or not. There were 50 or more valid cases, a case-to-variable ratio of at least 5–1, two or more correlations of 0.30 or higher, a sampling adequacy measure of at least 0.50 for each variable, an overall sampling adequacy measure of at least 0.50, and a probability of the Bartlett test of sphericity less than the level of significance for each variable.

### Ethics approval and consent to participate

We obtained ethical approval from the Amhara region public health institute and Institutional review board (IRB) of the University of Gondar. We received permission from Debre Tabor and Woreta towns’ health department administrat office. All methods were carried out in accordance with the Helsinki declaration guidelines and regulations. Before we want to collect the data, participation information sheet read for all participants and we obtained written informed consent from all participants. The participants were told that participation is fully voluntary and they had the right to refuse, withdraw at any time and refrain from answering any question that they don’t want to without any reprisal. Information gathered from the participants was stored in a secured cabinet by the principal investigator. Besides these, the data obtained from participants kept confidential. Those women who were expressing sever suicidal ideation referred to Debre Tabor referral Hospital psychiatry unit for further diagnosis and appropriate management. The decision for referral was made by the principal investigator based on the review of all women who were expressing suicidal ideation.

## Result

The detailed recruitment profile of women showed in the flow chart in Fig. 1. Between June 2019 and October 2019, 970 pregnant women were eligible to participate. Of this thirty-two (3.3%), declined to participate because of refusal to participate, and moving from the area. Finally, 938 women were included in the analysis, with a response rate of 96.7%. Our study revealed that the cumulative incidence of inadequate ANC visits was 62.58%. Of 938 participants, 858 (91.47%) women attended at least one antenatal care (ANC). However, from those who attended antenatal care services, one-fourth of 173 (20.16%) participants made their first visit during their first trimester. Of these, only 351(37%) of women had four or more ANC visits during their last antenatal period. The mean (± SD) of ANC visits was 3.1 ± 1.3, ranging from 0 to 6.

### Socio-demographic characteristics of the study participants

Out of the 938 mothers, 403 (43%) of them were found between the ages of 25–29 years. The mean (± SD) age was 27.1 ± 4.8 years. In this study, women who were married was 860 (92%). Regarding the level of education, 797 (85%) of the participants had formal education (Table 1).

**Table 1:** Frequency distribution of Socio-demographic factors among pregnant women at Debre Tabor and Woreta towns, Northwest Ethiopia, 2020.

Characteristics	Frequency (n = 938)	(%)
Age group		
≤ 19	34	3.63
20–24	238	25.37
25–29	403	42.96
30–34	179	19.08
> 35	84	8.96
Religion		
Orthodox	869	92.64
Muslim	56	5.97
Protestant	13	1.39
Ethnicity		
Amhara	926	98.72
Tigre	12	1.28
Education		
Unable to read and write	121	12.91
Able to read and write	20	2.13
Primary	241	25.69
High school	262	27.93
Diploma and above	294	31.34
Occupation		
Housewife	483	51.49
Employee	236	25.16
Merchant	148	15.78
Student	22	2.35
Daily laborer	49	5.22
Marital status		
Single	78	8.32
Married	860	91.68
Lack of food or hunger		
Yes	78	8.32
No	860	91.68
Debit		
Yes	95	10.13
No	843	89.87
Wealth index		
Low	314	33.47
Middle	313	33.37
High	311	33.16

### Obstetric and clinical characteristics of the study participants

The majority of the participants, 858 (91.47%) had one or more ANC visits during their last pregnancy. The result of this study revealed that 109 (12%) of the study population experienced current pregnancy complications. Among the participants, 160 (19%) did not have enough time to visit the ANC service. Around one-fifth of women 182 (19.40%) spent less than thirty minutes to arrive at the health facility (Table 2).

**Table 2:** Frequency distribution of obstetric and clinical factors among pregnant women at Debre Tabor and Woreta towns, Northwest Ethiopia, 2020.

Characteristics	Frequency (n = 938)	(%)
Depression		
Yes	326	34.75
No	612	65.25
Unplanned pregnancy		
Yes	354	37.74
No	584	62.26
Number of live children(529)		
One	252	47.64
Two-four	263	49.72
Five and above	14	2.64
Previous pregnancy complication		
Yes	116	20.60
No	447	79.40
History of current pregnancy complication		
Yes	109	11.62
No	829	88.38
Type of current pregnancy complication (n = 109)		
Anemia	35	32.11
APH	21	19.27
Edema	21	19.27
Hypertension	14	12.84
UTI	18	16.51
History of abortion(n = 563)		
Yes	94	16.70
No	469	83.30
Modes of previous abortion (n = 94)		
Spontaneous	84	89.36
Assisted	10	10.64
Stillbirth		
Yes	5	0.53
No	933	99.47
Gravidity		
One	375	39.98
Two-four	497	52.98
Five and above	66	7.04
Previous history of depression		
Yes	258	27.51
No	680	72.49
Family history of depression		
Yes	129	13.75
No	809	86.25
Parity (548)		
One	253	46.17
Two-four	277	50.55
Five and above	18	3.28
History of stillbirth (n = 563)		
Yes	36	6.39
No	527	93.61
Fear of pregnancy complication		
Yes	491	52.35
No	447	47.65
Chronic illness		
Yes	67	7.14
No	871	92.86
Types of chronic illness (67)		
Anemia	12	17.91
CHF	19	28.36
Hypertension	22	32.84
UTI	14	20.89
Lack of time for ANC visit		
Yes	160	18.65
No	698	81.35
Waiting a long time for ANC service		
Yes	576	67.13
No	282	32.87
Staff attitude influence ANC service		
Yes	752	80.17
No	186	19.83
Privacy influence ANC service		
Yes	652	75.99
No	206	24.01
Integration of ANC influence ANC service		
Yes	652	75.99
No	206	24.01
Long travel time influences ANC visit		
Yes	756	80.60
No	182	19.40
Initiation of ANC visit		
Early initiation	173	20.16
Late initiation	685	79.84

### Psychosocial and behavioral characteristics of the study participants

Out of 938 study populations, around one-fifth experienced hazardous level use of alcohol, only 203(22%) had strong social support, and 366 (39%) had life-threatening events (Table 3).

**Table 3:** Frequency distribution of psychosocial and behavioral factors among pregnant women at Debre Tabor and Woreta towns, Northwest Ethiopia, 2020.

Characteristics	Frequency (n = 938)	(%)
Life-threatening events		
Yes	366	39.02
No	572	60.98
Social support		
Poor	417	44.46
Moderate	318	33.90
Strong	203	21.64
Inter partner violence(IVP)		
Yes	493	52.56
No	445	47.44
Alcohol use		
Yes	206	21.96
No	732	78.04

### Factors associated with inadequate ANC visits

Antenatal depression, previous pregnancy complication, marital status, life-threatening events, history of depression, intimate partner violence, family history of depression, unplanned pregnancy, Social support, alcohol use, hunger, debt, long travel time for ANC visit, and late initiation of ANC visit all had a bivariate relationship with inadequate visits with the *P*-value < 0.20. All these variables were a candidate for multivariable analysis.

Multivariable logistic regression analyses showed that, after adjustment for covariates, women with antenatal depression symptoms (AOR = 1.96 (95% CI 1.220, 3.162)) were twice more likely to be inadequate ANC visits than women without antenatal depression symptoms. In addition, women with a history of late initiation of ANC visits (AOR = 2.20 (95% CI 1.393, 3.471)) and long travel time for ANC visits (AOR = 1.83 (95% CI 1.166, 2.870)) were increased odds of inadequate ANC visits as compared with their counterpart (Table 4).

**Table 4:** Bivariate and multivariable analysis for factors associated with inadequate ANC visits among women at Debre Tabor and Woreta towns, Northwest Ethiopia, 2020.

Characteristics	Inadequate ANC visit		COR at 95%CI	AOR at 95%C
	Yes	No		
Depression				
Yes	244	82	2.33 (1.734, 3.140)	1.96 (1.220,3.162) *
No	343	269	1	1
A complication of previous pregnancy				
Yes	82	34	1.64 (1.055, 2.554)	1.23 (0.752,2.021)
No	266	181	1	
Marital status				
Single	62	16	2.47 (1.403, 4.357)	2.48 (0.976,6.292)
Married	525	335	1	
Life-threatening events				
Yes	258	108	1.76 (1.334, 2.332)	0.93 (0.569,1.524)
No	329	243	1	
History of depression				
Yes	188	70	1.89 (1.382, 2.589)	1.44 (0.821,2.530)
No	399	281	1	
Intimate partner violence (IPV)				
Yes	332	161	1.54 (1.178, 2.005)	0.89 (0.578,1.370)
No	255	190	1	
Family history of depression				
Yes	97	32	1.97 (1.292, 3.015)	1.01(0.508,2.001)
No	490	319	1	
Unplanned pregnancy				
Yes	238	116	1.38 (1.048, 1.822)	1.18 (0.784,1.786)
No	349	235	1	
Social support				
Poor	278	139	1.17 (0.826, 1.663)	0.81(0. 464,1.422)
Moderate	181	137	0.77 (0.539, 1.111)	0.81 (0.482,1.369)
Strong	128	75	1	
Alcohol use				
Yes	147	59	1.65 (1.181, 2.315)	1.63 (0.997,2.661)
No	440	292	1	
Hunger				
Yes	55	23	1.47 (.889, 2.445)	0.88 (0.318,2.411)
No	532	328	1	
Debit				
Yes	70	25	1.77 (1.096, 2.845)	1.30 (0.479,3.528)
No	517	326	1	
Long travel time for ANC visit				
Yes	499	257	2.07 (1.495, 2.877)	1.83 (1.166,2.870) *
No	88	94	1	
Time of initiation of ANC visit				
Late initiation	430	255	2.10(1.500,2.946)	2.20 (1.393,3.471) **
Early initiation	77	96	1	

*NB* Abbreviations, *CI* confidence interval, *OR* odds ratio, *COR* crude odds ratio; *AOR* adjusted odds ratio, *ANC* antenatal care; ** (*P* ≤ 0.01); *(*P* < 0.05); Hosmer–lemeshow: 0.5411.

In a fully adjusted model; unplanned pregnancies (AOR = 1.18 (95% CI 0.784, 1.786)), the experience of threatening life events (AOR = 0.93 (95% CI 0.569, 1.524)), complication of previous pregnancy (AOR = 1.23 (95% CI 0.752, 2.021)), and intimate partner violence (AOR = 0.89 (95% CI 0.578, 1.370)), marital status (AOR = 2.48(95%CI 0.976, 6.292)), debit(AOR = 1.30 (95%CI 0.479, 3.528)), hunger (AOR = 0.88 (95%CI 0.318, 2.411)), family history of depression (AOR = 1.01 (95%CI 0.508, 2.001)), and history of depression (AOR = 1.44 (95% CI 0.821, 2.530)) during pregnancy were no longer associated with inadequate ANC visits, even if there were strongly associated in bivariate analyses. A Hosmer–Lemeshow test indicated that the model fit the data well (*p* = 0.5411).

## Discussion

The main finding was antenatal depression has effect on ANC visits in the Ethiopian urban settings. This is the first to our knowledge population-based, prospective cohort study examining specifically the effect of antenatal depression on ANC visits in our country. After controlling for known risk factors for inadequate ANC visits, antenatal depression (AOR = 1.96 (95% CI 1.220, 3.162)) remained significantly associated with inadequate ANC visits. The incidence of inadequate ANC visits in the exposed group due to antenatal depression was 25.33%.

Similarly, the positive effect of antenatal depression on inadequate ANC visits was reported in the United States of America, England, and East Asia region^22,23,25,26^. The possible explanation might be depression symptoms have loss of interest, and fatigue that disturb normal mother-infant bonding^43,44^. However, the association that we have reported about the increased odds of inadequate ANC visits among women with antenatal depressive symptoms did not replicate the findings of the recent cohort study from Ghana and the US of America^21,27^. These conflicting results might arise due to differences in socio-cultural, demographic factors, and sample size.

Furthermore, results from multivariable logistic regression analyses indicated that long travel time to health facility found to be highly influential over the use of ANC visits. In this study, women who travel more than 30 min to health facilities were 1.83 times more likely to have inadequate ANC visits as compared to their counterparts. This finding is supported by several other studies including in Ethiopia^45–54^. It is a fact that the accessibility of a health facility is the main barrier to maternal health service. However, our finding is in contrast to the study finding in Zambia^55^. The possible explanation might be due to Geographic, socio-cultural, and level of awareness differences.

Study findings from Cameroon and South Africa revealed that timely initiation of ANC visits is a key method to decrease pregnant women’s morbidity and mortality^3,56^. Also, pregnant women who received an early ANC check-up were much more likely to receive four or more WHO-recommended ANC visits^57^. Despite this importance, the results of this study revealed that only 173 (20.16%) of pregnant women were found to early initiate ANC visits. This estimate reflects under-utilization of ANC, and this could contribute to high maternal mortality. Those pregnant women who were late initiated ANC visits were 2.2 times more likely to have inadequate ANC visits as compared to their counterparts. The result was consistent with the research finding from Belgium^58^.

### Limitations

This study has limitations that prevent the full understanding of adequate antenatal care for ANC attendance in the study area; specifically, we did not address the content adequacy of antenatal care. Besides, we had no information on antidepressant medication for those referred to a psychiatric unit, thus it could not be assessed its roles as a confounder in multivariable analysis.

Despite these limitations, the results of this study have important implications. As this study confirmed that antenatal, depression has an adverse effect on ANC visits.

The health care providers and policymakers should think about the efforts to reduce the effect of antenatal depression such as routine screening of all pregnant women for depressive symptoms and treating them at the primary health care level by integrating the service; since screening is an effective approach for reducing morbidity in depressed people^59^, as a result, adequacy of ANC visits could be improved or maintained.

## Conclusions

This study suggests that antenatal depression affects ANC visits in Ethiopian urban settings. The incidence of inadequate ANC visits in the exposed group that is due to antenatal depression was 25.33%. Therefore, early detecting and treating depression during the antenatal period reduced significantly the impacts of depression on the health of the mother and fetus. Discussing antenatal depression and its effects on ANC visits for the community as a whole is warranted.

## Data Availability

All data generated or analyzed during this study are included in this published article.

## Abbreviations

ANC: Ante natal care
AOR: Adjusted odds ratio
CI: Confidence interval
COR: Crude odds ratio
OR: Odds ratio
CMD: Common mental disorder
EPDS: Edinburgh postnatal depression scale
FAST: Fast alcohol screening test
IPV: Intimate partner violence
LTE: List of threatening experiences
OSSS-3: Oslo-3 social support scale
